# Overexpression of an *Apocynum venetum* DEAD-Box Helicase Gene (*AvDH1*) in Cotton Confers Salinity Tolerance and Increases Yield in a Saline Field

**DOI:** 10.3389/fpls.2015.01227

**Published:** 2016-01-08

**Authors:** Jie Chen, Sibao Wan, Huaihua Liu, Shuli Fan, Yujuan Zhang, Wei Wang, Minxuan Xia, Rui Yuan, Fenni Deng, Fafu Shen

**Affiliations:** ^1^State Key Laboratory of Crop Biology, College of Agronomy, Shandong Agricultural UniversityTaian, China; ^2^College of Life Science, Shanghai UniversityShanghai, China; ^3^Cotton Research Institute – Chinese Academy of Agricultural SciencesAnyang, China; ^4^Cotton Research Center, Shandong Academy of Agricultural SciencesJinan, China

**Keywords:** DEAD-box helicase, *AvDH1*, cotton, salinity, yield, field trial

## Abstract

Soil salinity is a major environmental stress limiting plant growth and productivity. We have reported previously the isolation of an *Apocynum venetum* DEAD-box helicase 1 (*AvDH1*) that is expressed in response to salt exposure. Here, we report that the overexpression of *AvDH1* driven by a constitutive cauliflower mosaic virus-35S promoter in cotton plants confers salinity tolerance. Southern and Northern blotting analyses showed that the *AvDH1* gene was integrated into the cotton genome and expressed. In this study, the growth of transgenic cotton expressing *AvDH1* was evaluated under saline conditions in a growth chamber and in a saline field trial. Transgenic cotton overexpressing *AvDH1* was much more resistant to salt than the wild-type plants when grown in a growth chamber. The lower membrane ion leakage, along with increased activity of superoxide dismutase, in *AvDH1* transgenic lines suggested that these characteristics may prevent membrane damage, which increases plant survival rates. In a saline field, the transgenic cotton lines expressing *AvDH1* showed increased boll numbers, boll weights and seed cotton yields compared with wild-type plants, especially at high soil salinity levels. This study indicates that transgenic cotton expressing *AvDH1* is a promising option for increasing crop productivity in saline fields.

## Introduction

Salinity is a major abiotic stress limiting plant growth and productivity. It is estimated that at least 77 million ha of agricultural land is currently affected by salinity (Munns, 2002; Munns and Tester, 2008). Moreover, a rise in sea levels due to global warming is likely to increase these problems (Amin et al., 2012). Hence, it is necessary to obtain salinity-tolerant crop varieties. Many genes, including helicases, are known to be involved in salt stress tolerance (Hasegawa et al., 2000; Turan et al., 2012). Helicases belong to a class of molecular motor proteins in yeast, animals, and plants. DNA helicases unfold duplex DNA and are involved in replication, repair, recombination and transcription, whereas RNA helicases catalyze the unwinding of the secondary structures in RNA and are involved in transcription, ribosome biogenesis and translation initiation (Tuteja and Tuteja, 1996, 2004; Lüking et al., 1998). Most helicases are members of the DEAD-box protein superfamily and share a core region of highly conserved sequence motifs (Schmid and Linder, 1992; Pause et al., 1993).

To date, various helicase genes have been identified in several plants, and many of them are associated with diverse abiotic stresses, including temperature, light, oxygen, and salt stress (Owttrim, 2006, 2013; Vashisht and Tuteja, 2006). The *Arabidopsis* DEAD-box helicase, low expression of osmotically responsive genes 4, responds to low temperature (Gong et al., 2002, 2005). Two DEAD-box helicases, STRESS RESPONSE SUPPRESSOR1 and STRESS RESPONSE SUPPRESSOR2 function in abscisic acid (ABA)-dependent and ABA-independent abiotic stress signaling pathways in *Arabidopsis thaliana* (Kant et al., 2007). In addition, the functional involvement of a putative alfalfa helicase in the antioxidative responses of plants has also been reported (Luo et al., 2009). Recently, the overexpression of *PDH45*, a *Pisum sativum* helicase gene, has been shown to improve the salinity tolerance of transgenic rice (Sahoo et al., 2012; Gill et al., 2013; Nath et al., 2015) and sugarcane (Augustine et al., 2015). The overexpression of the rice mitochondrial helicase *OsSUV3* (suppressor of Var 3) resulted in greater salinity tolerance in rice without yield loss (Tuteja et al., 2013). Tuteja et al. (2014) also showed that the plants overexpressing the rice antigen-B-associated transcript 1 (*OsBAT1*) helicase gene had augmented salinity stress tolerance through the enhanced detoxification of reactive oxygen species (ROS) and the expression of stress-responsive genes. However, these studies of transgenic helicases plants in saline conditions were solely greenhouse-based. To measure yield traits and validate these greenhouse-based findings of improved salinity tolerance, saline field trials using transgenic plants were necessary (Flowers, 2004).

Cotton (*Gossypium hirsutum L.*) is an important fiber and oil crop grown worldwide. Although it is classified as a salt-tolerant crop, there are obviously varietal differences in response to soil salinity, and its growth and yield are severely affected in higher salinity soil (Ashraf, 2002). Therefore, it is of agricultural importance to improve the salt tolerance of cotton. Recently, a few reports have been published on the genetic engineering of salt tolerance in cotton using various genes. For example, the overexpression of an *Arabidopsis* vacuolar H^+^-pyrophosphatase gene, *AVP1*, in cotton improves drought and salt tolerance, and increases fiber yield in field conditions (Pasapula et al., 2011). An *Atriplex hortensis* choline monooxygenase gene, was found to be involved in glycine betaine synthesis and salinity tolerance in transgenic cotton lines (Zhang et al., 2009). However, to our knowledge, no report has been published on the genetic engineering of cotton using DEAD-box helicase proteins to improve salt stress tolerance.

We previously isolated and characterized *Apocynum venetum* DEAD-box helicase 1 (*AvDH1*; Accession number EU145588; Liu et al., 2008). This gene is expressed in response to NaCl but not polyethlene glycol or ABA (Liu et al., 2008). In this study, we overexpressed *AvDH1* in cotton to test whether cotton’s performance could be improved under salinity stress conditions both in the growth chamber and in a saline field.

## Results

### Regeneration and Analysis of Transgenic Cotton Plants

**FIGURE 1:**
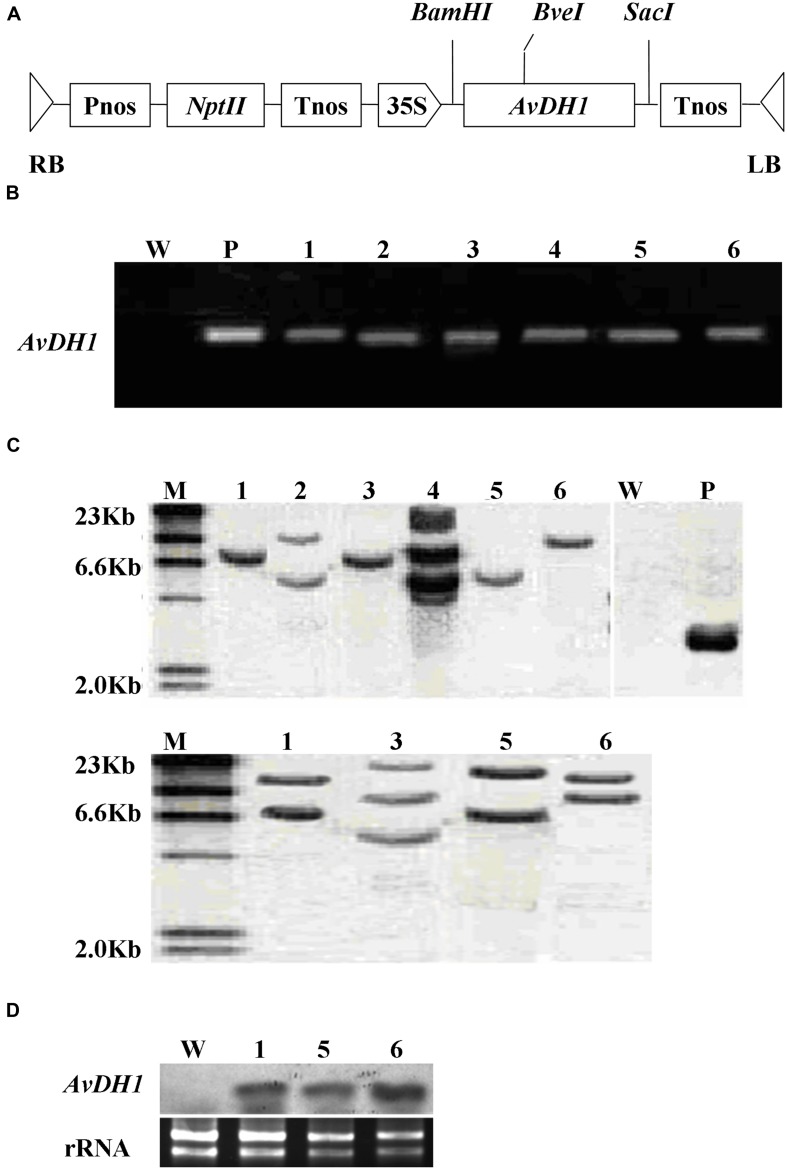
**Schematic structure of the T-DNA and molecular analysis of *AvDH1*-expressing cotton. (A)** T-DNA region of the vector pBI121-*AvDH1*. RB, right T-DNA border; LB, left T-DNA border; Pnos, nopaline synthase gene promoter; *nptII*, neomycin phosphotransferase gene; Tnos, nopaline synthase gene terminator; 35S, cauliflower mosaic virus 35S promoter; *AvDH1*, *Apocynum venetum* DEAD-box helicase gene. **(B)** PCR analysis of genomic DNA from an untransformed control (W) and six independent T_0_ transgenic lines (1–6). P, pBI121-*AvDH1* as positive control. **(C)** Southern blot analysis of *AvDH1*-transformed cotton lines. Genomic DNA was digested with *Stu*I (upper) and *Bve*I (lower). The membrane was hybridized with a [^32^P]-labeled *AvDH1* probe. M, molecular marker; P, pBI121-*AvDH1* as positive control; W, untransformed control; 1–6, transgenic lines 08-66, 08-89, 08-90, 08-92, 08-26, and 08-87, respectively. **(D)** Three independent T3 transgenic lines were confirmed by Northern blotting. Lower panel shows rRNA to confirm equal loading. 1, line 08-66; 5, line 08-26; 6, line 08-87.

Approximately 1,200 hypocotyl explants were infected with the *Agrobacterium tumefaciens* strain *LBA4404* containing pBI121-*AvDH1* (**Figure 1A**). Using our transformation protocol, 45 independently regenerated shoots were obtained. Well-grown shoots without roots were removed and grafted to wild-type rootstocks. More than 80% of the grafted shoots gave rise to plantlets. Six transgenic lines (of 36 putative transgenic lines generated) were selected based on their proficient growth on a kanamycin-supplemented medium. Additionally, these six transgenic lines were checked for transgene integration using PCR amplification of *AvDH1* from their genomic DNA (**Figure 1B**). A Southern blot of genomic DNA extracted from leaves showed that the transgene was incorporated into the cotton genome in the tested lines (**Figure 1C**). Moreover, the analysis illustrated that the plants 08-66 (**Figure 1C** upper, 1), 08-90 (**Figure 1C** upper, 3), 08-26 (**Figure 1C** upper, 5) and 08-87 (**Figure 1C** upper, 6) contained single transgene copies, whereas plants 08-89 (**Figure 1C** upper, 2) and 08-92 (**Figure 1C** upper, 4) had two and three copies, respectively, of the foreign gene. The different positions and numbers of the bands detected in the Southern blot indicated random integration events. The single-copy events in the four transgenic lines (08-66, 08-90, 08-26, and 08-92) were further confirmed by Southern hybridization of *Bve*I-digested DNA with the same probe, and two expected bands were observed in line 08-66 (**Figure 1C** lower, 1), 08-26 (**Figure 1C** lower, 5) and 08-87 (**Figure 1C** lower, 6), whereas three bands were found in line 08-90 (**Figure 1C** lower, 3). We deduced that line 08-90 might contain two copies integrated at the same insertion site (**Figure 1C**, 3). Each of the six positive transgenic plants represented an independent transformant. They were allowed to self-pollinate, and seeds were collected. Seeds from three T_0_ transgenic lines, 08-66, 08-87 and 08-26, which had single-copy transgene integration events, were used for a segregation analysis of the *nptII* kanamycin-resistance gene. The percentage of kanamycin-resistant progeny was 81% for line 08-66, 76% for line 08-26, and 75% for line 08-87, which is consistent with the expected Mendelian segregation of 75% for a single T-DNA insertion. The T_3_ generation lines were analyzed for *AvDH1* expression by Northern blotting, which revealed that line 08-26 (**Figure 1D**, 5) is a low expressing plant when compared with lines 08-66 (**Figure 1D**, 1) and 08-87 (**Figure 1D**, 6) that showed high expression levels of the transgene. When grown in the greenhouse, lines 08-66, 08-87, and 08-26 were phenotypically normal, and they also grew well in the field. The three lines were used in salt stress tolerance studies.

### Overexpression of *AvDH1* Enhanced Cotton Salt Tolerance in the Growth Chamber

**FIGURE 2:**
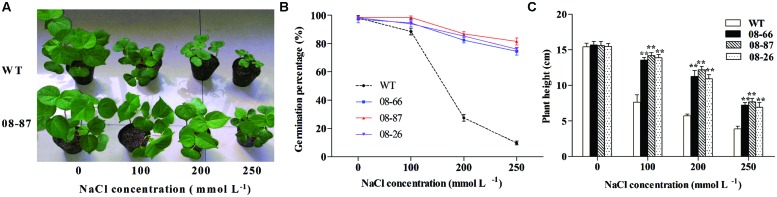
**Salinity tolerance of WT and transgenic cotton expressing *AvDH1* grown in a growth chamber. (A,C)** WT and transgenic plants were subjected to 14 days of salt stress after emergence (8 days after sowing) in a growth chamber. **(A)** Representative picture of WT and transgenic (08-87) seedlings. **(C)** Plant height of WT and transgenic lines 08-66, 08-87, and 08-26. Asterisks indicate a significant difference from the WT under the same salt treatment conditions at ^∗^*P* < 0.05 or ^∗∗^*P* < 0.01 as determined by a *t*-test. **(B)** Germination percentages of seeds sown in sand under irrigation with different concentrations of NaCl (0, 100, 200, and 250 mM) in Hoagland solution. The germination percentages were calculated 10 days later. Values are the means of three independent experiments ± SD.

Pot experiments in a growth chamber showed that transgenic cotton had a higher germination percentage than wild-type plants under salt stress (**Figure 2B**). Transgenic cotton lines expressing *AvDH1* and WT seedlings were morphologically similar when grown without NaCl (**Figure 2A**). In the presence of NaCl, WT seedlings showed stunted phenotypes, whereas the transgenic plants grew more vigorously (**Figure 2A**). Statistically similar results were obtained for the three tested transgenic lines (representative picture of 08-87 line). To determine the degree of NaCl tolerance, the plant heights of WT and *AvDH1* overexpressing transgenic seedlings under different concentrations of NaCl was measured. The increased NaCl tolerance manifested by the transgenic plants, compared with that of the WT plants, was apparent at each NaCl concentration. Under 250 mM NaCl, the plant heights of transgenic seedlings was 6.93–7.71 cm compared with 3.87 cm for the WT seedlings (**Figure 2C**). Similar results were obtained for all three transgenic lines, indicating that the lowest expression level was sufficient to confer salinity tolerance.

### Cell Membrane Ion Leakage, MDA Content, and SOD Activity under Salt Stress

**FIGURE 3:**
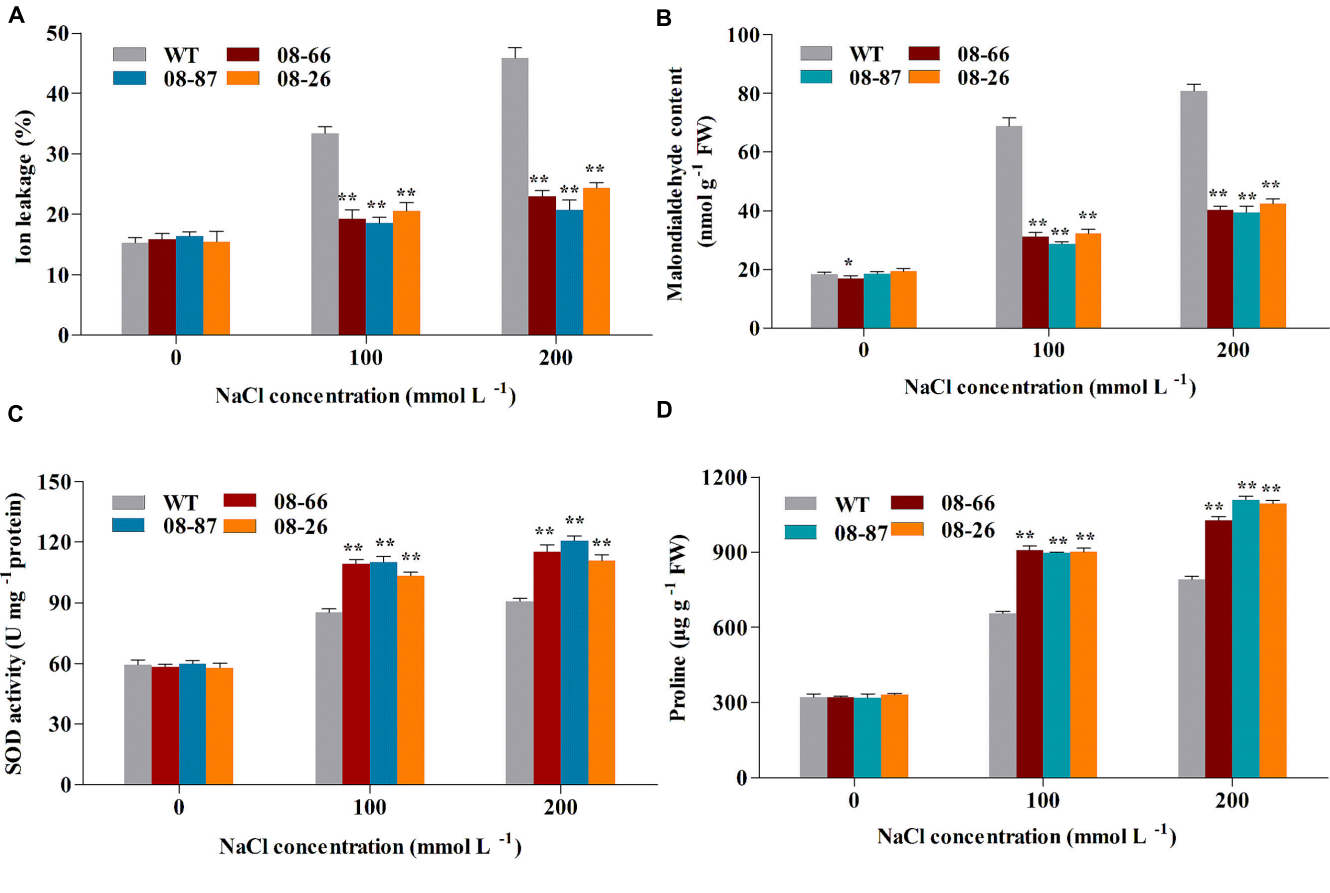
**Membrane ion leakage, MDA content, SOD activity and proline content of transgenic and wild-type cotton with or without stress treatments. (A)** Membrane ion leakage of transgenic and WT cotton. **(B)** MDA content of transgenic and WT cotton. **(C)** SOD activity of transgenic and WT cotton. **(D)** Proline content of transgenic and WT cotton. WT and transgenic plants were subjected to 14 days of salt stress from the four-leaf stage. Values are the means of three independent experiments ± SD. Asterisks indicate a significant difference from the WT under the same salt treatment conditions at ^∗^*P* < 0.05 or ^∗∗^*P* < 0.01 as determined by a *t*-test.

To study the effects of salt stress on cotton seedlings further, leaf cell membrane ion leakage, MDA content and superoxide dismutase (SOD) activity were determined in non-stressed plants and plants salt stressed for 14 days. As shown in **Figure 3**, there were no significant differences in ion leakage, MDA content and SOD activity between the WT and any of the transgenic lines under normal conditions. However, significant differences appeared in the ion leakage, MDA content and SOD activity between *AvDH1*-expressing lines and the WT under increased salt conditions. Ion leakage and MDA content increased with increasing salt concentrations in WT plants, while the cell ion leakage and MDA content of *AvDH1* lines changed little. Under 100 and 200 mM NaCl treatments, the ion leakage of *AvDH1* lines was 38–44% and 47–55% less than that of the WT, respectively (**Figure 3A**). The MDA content was 47–50% lower in the leaves of transgenic cotton compared with WT plants under 200 mM NaCl treatment (**Figure 3B**). An increase in the SOD activity was noted in transgenic lines and WT under all of the salinity treatments. The SOD activity of the transgenic lines was 21–29% and 22–33% more than that of the WT under the two salt treatments, respectively (**Figure 3C**).

### Proline, Na^+^ and K^+^ Contents under Salt Stress

**FIGURE 4:**
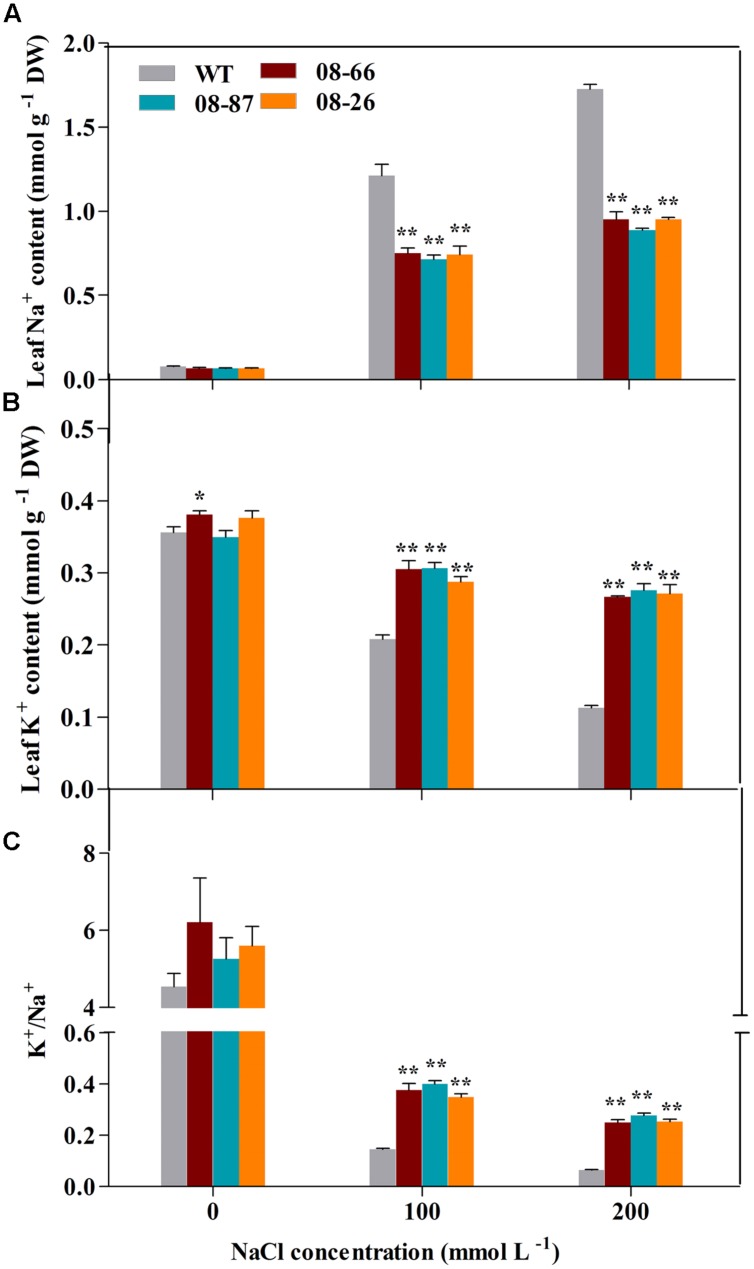
**Na^+^ and K^+^ ion content in the leaves of transgenic and wild-type cotton with or without stress treatments. (A)** Na^+^ content of transgenic and WT cotton. **(B)** K^+^ content of transgenic and WT cotton. WT and transgenic plants were subjected to 14 d of salt stress from the four-leaf stage. **(C)** K^+^/Na^+^ ratio of transgenic and WT cotton. Values are the means of three independent experiments ± SD. Asterisks indicate a significant difference from the WT under the same salt treatment conditions at ^∗^*P* < 0.05 or ^∗∗^*P* < 0.01 as determined by a *t*-test.

Proline, Na^+^ and K^+^ contents under non-stressed conditions were similar for transgenic and WT cotton (**Figures 3D and 4A,B**). However, transgenic cotton accumulated 37–39% and 30–40% more proline than WT cotton under 100 and 200 mM NaCl treatments, respectively (**Figure 3D**). Besides, transgenic plants accumulated more K^+^ and less Na^+^ than the WT plants (**Figures 4A,B**), and the K^+^/Na^+^ ratio was higher in the transgenic plants (**Figure 4C**).

### Transgenic *AvDH1* Cotton has an Increased Yield in a Saline Field

**FIGURE 5:**
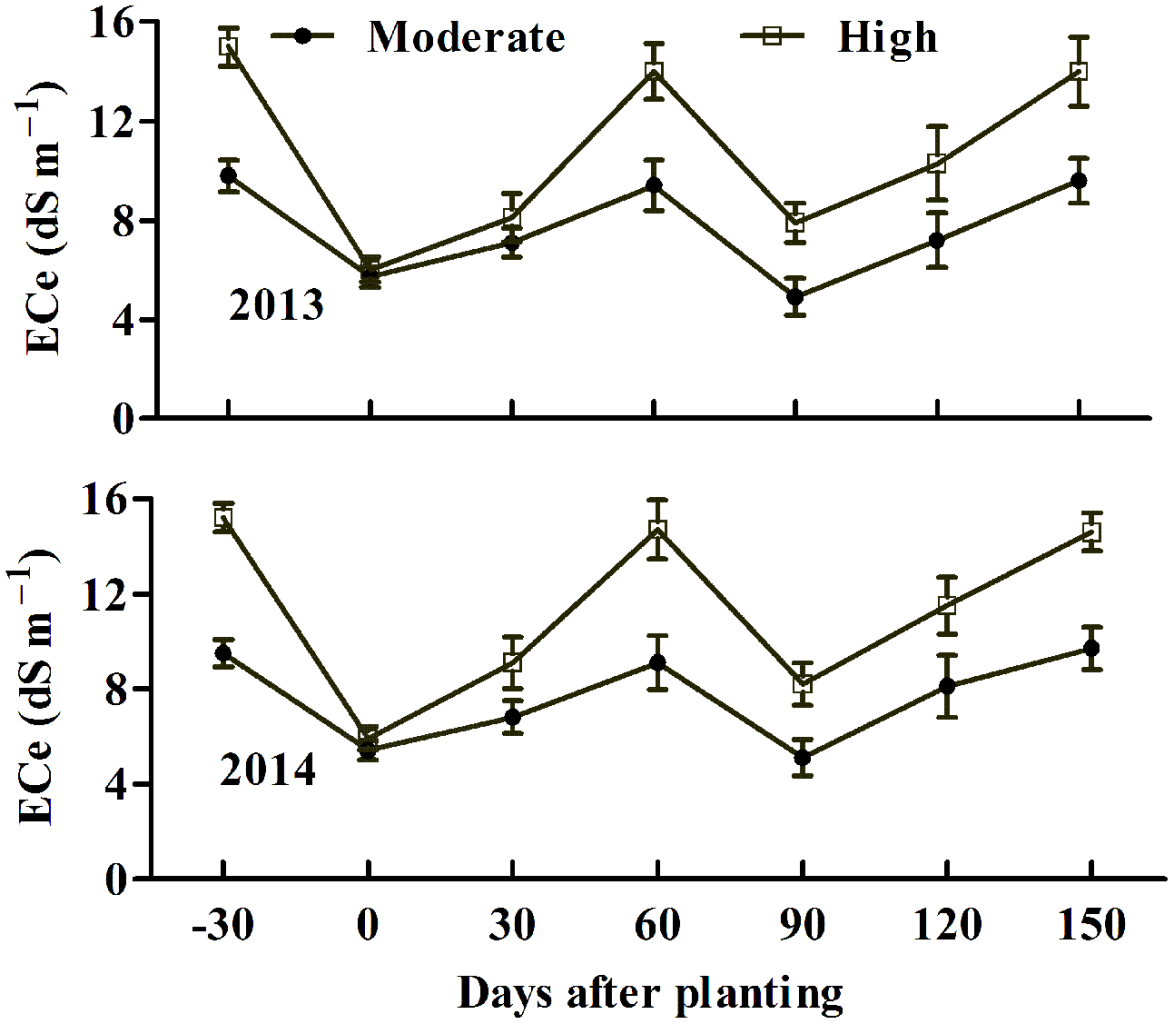
**Dynamics of soil salinity in the 20-cm surface soil layer at different days before and after sowing in 2013 and 2014.** Values at -30 and 0 days after planting showed ECe (electrical conductivity of soil saturated paste extract) before irrigation (without leaching) and at sowing (after leaching). For determination of salinity, three surface (0–20 cm) soil samples per subplot were collected at a 30-days interval. All of the samples were air-dried, ground and passed through a 2-mm sieve. Saturated soil extracts of the samples were prepared following the procedure described previously (Richards, 1954). ECe was then measured with a conductivity meter. Vertical bars show ± SD.

**FIGURE 6:**
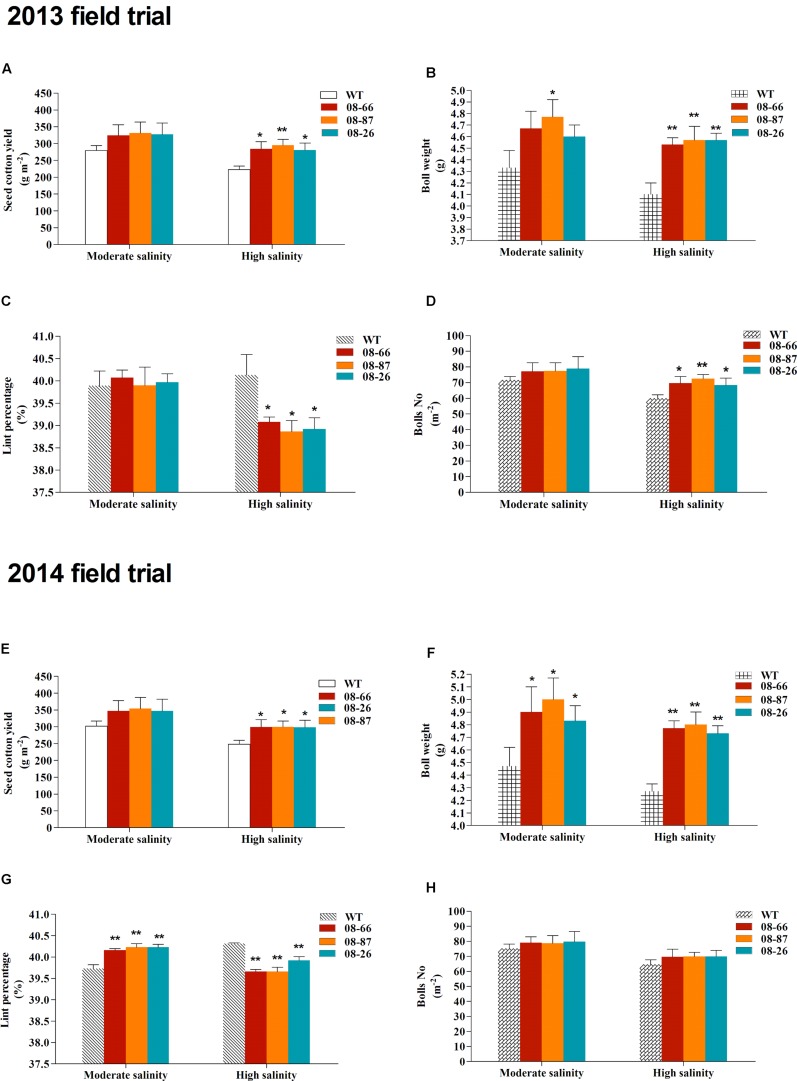
**Field trials in 2013 and 2014.** The seed cotton yield **(A,E)**, boll weight **(B,F)**, lint percentage **(C,G)** and number of bolls **(D,H)** of transgenic cotton expressing *AvDH1* (08-66, 08-87, and 08-26) and WT in a moderate- and high-salinity field area. Values are the mean ± SD. Asterisks indicate a significant difference from the WT under the same salt treatment conditions at ^∗^*P* < 0.05 or ^∗∗^*P* < 0.01 as determined by a *t*-test.

Field trials were conducted in cropping years 2013 and 2014 in two nearby saline fields. The soil salinity and fertility of the two experimental fields are shown in **Table 1**. Transgenic cotton plants expressing *AvDH1* (08-66, 08-87, and 08-26) and WT cotton (Lu 613) plants were used in these experiments. The dynamics of salinity in the plant root zone (0–20 cm) at different days before and after sowing was determined (**Figure 5**). Transgenic cotton in the saline field exhibited higher germination percentages compared with wild-type plants (**Table 2**). As expected, the three transgenic lines produced statistically higher average boll numbers, boll weights, and seed cotton yields than those of WT plants under both moderate and high soil salinity levels (**Figure 6**). In the field trial of 2013, in the moderate-salinity area, transgenic line 08-87 had a significantly higher (10.2%) boll weight than the WT plants (**Figure 6B**). In the high-salinity area, the average seed cotton yields of transgenic *AvDH1* cotton plants were significantly higher (26.2–32.5%) than that of the WT (**Figure 6A**). The boll numbers and boll weights of transgenic cotton expressing *AvDH1* were significantly greater (14.2–20.9% and 10.5–11.5%, respectively) than those of WT plants (**Figures 6B,D**). However, the lint percentage of the *AvDH1* lines was significantly lower (2.6–3.2%) than that of WT plants in the high-salinity area (**Figure 6C**).

**Table 1:** The soil characteristics and salinity of the two experimental fields.

Salinity level	ECe (dS m^-1^)	pH	Organic matter (g kg^-1^)	Available N (mg kg^-1^)	Available P (mg kg^-1^)	Available K (mg kg^-1^)
Moderate	9.8 ± 0.4^1^	8.1 ± 0.2	11.2 ± 0.8	50.2 ± 4.6	16.2 ± 2.1	152.2 ± 11
High	15.6 ± 0.6	8.5 ± 0.2	10.9 ± 1.1	49.5 ± 4.2	16 ± 1.9	151.7 ± 12

*ECe, electrical conductivity of soil saturated paste extract. ^1^Data (mean ± SD) were collected from 0- to 20-cm soil depths in the early spring before irrigation and leaching in 2013*.

**Table 2:** Seed germination of transgenic cotton expressing *AvDH1* (08-66, 08-87, and 08-26) and WT in a moderate- and high-salinity field area in 2013 and 2014.

Line	2013 Emergence rate (%)		2014 Emergence rate (%)	
	Moderate	High	Moderate	High
WT	70.4 ± 1.88	51.2 ± 1.95	69.0 ± 2.27	49.8 ± 1.08
08-66	76.3 ± 2.26^∗^	68.5 ± 0.78^∗∗^	77.3 ± 3.70^∗∗^	70.5 ± 0.71^∗∗^
08-87	80.2 ± 1.45^∗∗^	74.5 ± 1.63^∗∗^	80.1 ± 1.29^∗∗^	72.1 ± 2.31^∗∗^
08-26	78.3 ± 0.81^∗∗^	71.9 ± 2.40^∗∗^	76.4 ± 2.47^∗^	68.6 ± 3.13^∗∗^

*The germination rate was counted 15 days after sowing. Values are the mean ± SD. Asterisks indicate a significant difference from the WT under the same salt treatment conditions at ^∗^P < 0.05 or ^∗∗^P < 0.01 as determined by a t-test.*

In our field trial of 2014, we obtained similar results. In the moderate-salinity area, transgenic plants had significantly higher (8.1–11.9%) boll weights than the WT (**Figure 6F**). What’s more, the lint percentages of the transgenic plants were significantly higher (1.1–1.3%) than that of WT plants (**Figure 6G**). In the high-salinity area, plant growth was reduced, however, the *AvDH1* transgenic cotton had a significantly greater survival rate than the WT plants (**Figures 6A,B**). WT plants exhibited the highest mortality at the end of the season compared with the transgenic plant lines (**Figure 6B**). The average seed cotton yields of the *AvDH1* lines were significantly higher (20.4–20.9%) than that of WT plants (**Figure 6E**). The boll weights in transgenic *AvDH1* lines were significantly greater (10.8–12.4%) than that in the WT (**Figure 6F**), whereas the lint percentages of the *AvDH1* lines were significantly lower (0.97–1.6%) than that of WT plants in the high-salinity area (**Figure 6G**).

## Discussion

Since salinity is a major abiotic stress affecting plant productivity worldwide, improving salt tolerance in crops became an important objective in agriculture. The transfer of salt-inducible genes to impart salt tolerance in host plants by genetic engineering is a powerful approach for increasing tolerance to salt stress (Munns, 2005; Waditee et al., 2007). Helicases have been found to play key roles in response to various abiotic stresses, including temperature, light, oxygen and salt stress (Owttrim, 2006, 2013; Vashisht and Tuteja, 2006). Previously, we cloned a salt responsive DEAD-box helicase gene, *AvDH1*, from the halophyte dogbane. The study here was undertaken to evaluate the function and potential of *AvDH1* in conferring salinity stress tolerance in cotton.

In this study, three confirmed independent transgenic lines (08-66, 08-87, and 08-26) were carried forward for functional validation under salinity stress. Our results showed that *AvDH1* overexpression in cotton confers salinity tolerance. Transgenic cotton seedlings expressing *AvDH1* exhibited better growth than WT plants (**Figure 2A**) and had greater plant heights (**Figure 2C**) under increased NaCl concentrations, indicating the positive influence of the transgene on the overall well-being of the plant. No statistical difference was found among the three transgenic lines with varied *AvDH1* expression levels (**Figures 2A,C**), indicating that the lowest expression level was sufficient to confer salinity tolerance. Additionally, no growth differences between non-transgenic and transgenic cotton expressing *AvDH1* (08-66, 08-87, and 08-26) in non-saline conditions were seen. This is in agreement with previous studies, where transgenic helicase plants and WT were morphologically similar when grown without NaCl (Gill et al., 2013; Tuteja et al., 2013, 2014; Augustine et al., 2015).

Previous studies of transgenic helicases in plants under saline conditions were solely greenhouse-based (Sanan-Mishra et al., 2005; Amin et al., 2012; Sahoo et al., 2012; Tuteja et al., 2014), and there are no reports on saline field trials evaluating the growth and yield of transgenic plants expressing a helicase gene. In this study, the growth of transgenic cotton expressing *AvDH1* was evaluated under saline conditions not only in a growth chamber but also in a saline field trial under moderate and high soil salinity levels. The transgenic cotton expressing *AvDH1* showed an increase in boll numbers, boll weights and seed cotton yields compared with WT plants in our field trial, especially under high soil salinity levels (**Figure 6**). Averaged across 2 years, the transgenic lines 08-66, 08-87, and 08-26 produced 15.4, 17.9, and 15.9%, respectively, more seed cotton than WT in the moderate-salinity area and 23.9, 26.4, and 23.1%, respectively, in the high-salinity area (**Figures 6A,E**). An increase in boll numbers and boll weights contributed toward the increase in seed cotton yields of the transgenic *AvDH1* cotton lines (**Figure 6**). This increase in cotton yield supports the growth chamber experimental results presented in this study. Notably, the lint percentage of the transgenic lines was significantly lower than that of WT under high-salinity stress (**Figures 6C,G**). It was reported that the ginning out-turn increased with increasing concentrations of salt, however, the salt-tolerant lines had lower ginning out-turns than that of the salt-sensitive lines (Ashraf and Ahmad, 2000). Our results indicated that the transgenic *AvDH1* cottons were more salt tolerant compared with WT plants. We deduced that salinity had less effects on fiber development than seed development, and large-seeded *AvDH1* lines had lower lint percentages than small-seeded WT plants. Additionally, our recent work showed that DEAD-box helicase genes in *Gossypium raimondii* are highly expressed at the fiber initiation stage (Chen et al., 2014). The *AvDH1* gene may also be expressed at the initiation stage of cotton fiber development and influence the lint percentage under high-salinity stress, which needs to be studied in detail in the future.

Salt stress can induce the rapid accumulation of ROS, which can cause damage to cellular macromolecules, ultimately affecting membrane stability (Apel and Hirt, 2004; Gill and Tuteja, 2010; Gill et al., 2012). Therefore, improving the ROS scavenging capacity is vital for plants to resist salt-stress conditions. SOD is an important ROS scavenging enzyme integral to plant stress tolerance (Lamb and Dixon, 1997; Mittler et al., 2004). In this study, the *AvDH1* transgenic lines showed lesser ion leakage, lower level of MDA along with increased SOD activity (**Figures 3A–C**). Therefore, we deduced that the elevated SOD activity in *AvDH1* transgenic lines may result in decreased ROS levels, preventing membrane damage and increasing plant survival rates. Consistent with our results, previous studies have shown that the overexpression of helicase genes could elevate SOD activity (Gill et al., 2013; Tuteja et al., 2013; Banu et al., 2014). Present study also showed that under salt stress, *AvDH1*-overexpressing cotton accumulated more proline than WT plants (**Figure 3D**). Proline accumulation frequently correlates with tolerance to salt stress in plants (Ashraf and Harris, 2004; Ben et al., 2008). Besides, transgenic cottons accumulated more Na^+^ and less K^+^ and maintained a higher K^+^/Na^+^ ratio than the WT plants did (**Figure 4**). It is well-established that salt-tolerant cultivars have more K^+^ and less Na^+^ to maintain a higher K^+^/Na^+^ ratio (Morant-Manceau et al., 2004).

The exact mechanism of helicase-mediated salinity tolerance is not well-understood. Gill et al. (2013) suggested that the exact mechanism of *PDH45*-mediated salinity stress tolerance in rice could be due to the positive regulation of the antioxidant machinery. Tuteja et al. (2014) reported that the salinity stress tolerance in *OsBAT1* transgenic rice plants might be due to the increased detoxification of ROS and interactions of *OsBAT1* with components of different signaling pathways. We hypothesize that *AvDH1* may be involved in salt tolerance by improving the antioxidant machinery and by maintaining the genome integrity of the transgenic cotton plants under salt stress conditions. We provide direct evidence for the involvement of a DEAD-box helicase (*AvDH1*) in conferring salinity tolerance in transgenic cotton plants. Our results indicate that transgenic cotton expressing *AvDH1* is a promising option for increasing crop productivity in saline fields. However, further studies are needed to investigate the precise mechanism of salinity tolerance in cotton mediated via the *AvDH1* helicase.

## Materials and Methods

### Vector Construction

The full-length coding sequence of *AvDH1* (GenBank accession number EU145588) amplified using the primers AVP1 (5′-CGGGATCCATGGCTACTCCTACTTCTGG-3′) with a *Bam*HI site (underlined) and AVP2 (5′-CGAGCTCGCCAAAAACCCTAAACTATCAC-3′) with a *Sac*I site (underlined) was inserted into the corresponding sites behind the cauliflower mosaic virus-35S promoter in the pBI121 binary vector. This binary plasmid carries a selectable marker gene, *nptII*, under the nos promoter. The ligated construct (pBI121-*AvDH1*) was introduced into *A. tumefaciens* (strain *LBA4404*) using the freeze-thaw method (Hofgen and Willmitzer, 1988).

### Cotton Transformation

Cotton (*G. hirsutum L*. cv. lu 613) seeds were dehusked manually and sterilized with 0.1% (w/v) HgCl_2_ for 5 min followed by four washes with sterile distilled water. Seeds were germinated on Murashige and Skoog (MS) medium containing 2% (w/v) glucose, pH 5.8. Hypocotyl segments (5–6 mm) excised from 5 to 7 days-old seedlings were used for transformations. The hypocotyl segments were used directly or precultured for 2 days on hormone-free MS medium supplemented with B5 vitamins prior to infection and cocultivation with bacterial cultures. The explants were infected with *A. tumefaciens LBA4404* containing pBI121-*AvDH1* in the bacterial suspension for ∼20 min, and blotted dry on sterile filter paper to remove excess *A. tumefaciens*. They were transferred to basal medium and cocultivated under dark conditions for 48 h at 22 ± 1°C. After coculturing, the explants were washed with sterile distilled water, blotted dry and transferred to a callus-induction medium containing basal MS, 0.1 mg L^-1^ kinetin, 0.1 mg L^-1^ NAA, 3% glucose, 1 g L^-1^ MgCl_2_, 450 mg L^-1^ cefotaxime, and 95 mg L^-1^ kanamycin. Pre-embryogenic calli were induced ∼3–4 weeks after incubation and transferred onto fresh medium for further selection until a healthy growing calli were obtained. The embryogenic calli were excised and transferred to glass flasks containing a growth regulator-free callus medium containing additional 250 mg L^-1^ activated charcoal and 100 mg L^-1^ kanamycin for cellular proliferation. The healthy calli were transferred to embryo differentiation medium containing MS salts supplemented with B5-vitamin, 1.9 g L^-1^ KNO_3_, 0.85 g L^-1^ MgCl_2_, 0.5 g L^-1^ asparagine, 300 mg L^-1^ cefotaxime, 2% (w/v) glucose, 0.3% (w/v) Phytagel and 100 mg L^-1^ kanamycin, and maintained on this medium until somatic embryos developed and geminated. Shoot regeneration occurred within 9–10 weeks. Selected well-grown shoots with true leaves without roots were cut off and grafted to rootstocks in the greenhouse. Thirty batches of transformation experiments were performed (40 explants each) to confirm the reproducibility of the protocol.

### PCR and Southern Blot

Genomic DNA was isolated from the young leaves of transgenic and WT plants using a Plant Genomic DNA Kit (*Tiangen Biotech.*, China). The PCR amplification was performed with *AvDH1*-specific primers. The forward and the reverse primer sequences were 5′-GCTCCTACAAATGCCATCATTGC-3′ and 5′-GATAGTGGGATTGTGCGTCATCCC-3′, respectively. The predicted PCR product was 225 bp in length. For Southern hybridization, cotton genomic DNA (15 μg) was digested with *Stu*I and *Bve*I separately, electrophoresed and blotted on Hybond N membranes (*Amersham Pharmacia*). A [^32^P]-labeled *AvDH1* gene was used as the probe. Southern blots were hybridized by following the standard procedure provided by the manufacturer. After hybridization and stringent washing, the radioactive membranes were exposed to an imaging plate (*Fuji Photo Film*, Japan) for 5 h or overnight to record the images.

### Northern Analysis

Total RNA was extracted using the RNeasy plant mini kit (*Qiagen*, Fremont, CA, USA) according to the manufacturer’s instructions. Approximately 30 μg of total RNA samples resolved on a 1.5% formaldehyde-agarose gel were transferred to Hybond N membranes (*Amersham Pharmacia*) and probed as described above.

### Salt Treatment in the Growth Chamber

Seeds of transgenic lines and the WT were sown in 30 cm-diameter flowerpots filled with clean sand to determine the germination frequency of the seeds. There were 25 seeds per pot and 16 replicate pots for each line. The pots of each line were randomly divided into four groups and watered twice a week with Hoagland solution supplemented with 0, 100, 200, or 250 mM NaCl. Seeds were considered to have germinated when their coleoptiles appeared within 10 days after sowing.

To determine the tolerance of seedlings to NaCl stress, seeds of the WT and transgenic cotton, the T_4_ generation of independent lines 08-66, 08-87 and 08-26, were sown in 10 cm-diameter plastic pots filled with clean sand and grown in a growth chamber at 28/20°C (day/night) with a photon flux density (PFD) of 500 μmol m^-2^ s^-1^, a relative humidity of 60–70% and a photoperiod of 14/10 h (light/dark). There were 15 pots (replicates) for the WT and each transgenic cotton line. Salt treatments were performed after emergence (8 days after sowing) by irrigation twice a week with Hoagland’s solution containing increasing concentrations of NaCl (0, 100, 200, and 250 mM). WT and each transgenic line cultured in Hoagland’s solution were used as controls. Photographs were taken 14 days after the initiation of the stress treatment and at that time plant heights were measured. In another assay of salt tolerance, seedlings of WT and transgenic cotton were grown in 30 cm-diameter plastic pots filled with clean sand and watered with Hoagland’s nutrient solution twice a week. At the four-leaf stage, seedlings of the WT and each transgenic line were exposed to salinity by adding NaCl to the nutrient solution in 50 mM increments every 24 h, until the final concentrations of 100 and 200 mM were reached. Seedlings of the WT and each transgenic line cultured in Hoagland’s solution were also used as controls. After 14 days of salt treatment, SOD activity, ion leakage, and contents of proline, malondialdehyde (MDA) and Na^+^ and K^+^ in leaves were measured. Each parameter was measured in four replicates.

### Measurement of Ion Leakage

The ion leakage was determined by measuring the conductivity. Ion leakage expressed as a percentage was calculated as described previously (Ai et al., 2008). Segments (1 cm^2^) were obtained from cotton cotyledons for the measurements. Electrolyte leakage was calculated using the following formula: electrolyte leakage (%) = L_t_/L_0_ × 100, where the conductivity measurements L_t_ and L_0_ corresponded to the plant leaves before and after boiling in water, respectively.

### Assays of SOD Activity

The SOD activity was measured according to the method described by Giannopolitis and Ries (1977). Briefly, 3 mL of reaction mixture containing 50 mM potassium phosphate (pH 7.8), 0.1 mM EDTA, 13 mM methionine, 75 μM nitro-blue tetrazolium (NBT), 2 μM riboflavin and 150 μL of enzyme extract was illuminated at 30–35°C with a light intensity of 200 μmol m^-2^ s^-1^ for 10 min, and the absorbance was measured at 560 nm. The protein content of the extract was determined as described by Bradford (1976) using bovine serum albumin as the standard. One unit of SOD activity was defined as the amount of enzyme required to inhibit 50% NBT photoreduction under assay conditions. SOD activity was expressed as unit mg^-1^ of protein.

### Proline, Malondialdehyde (MDA) and Na^+^ and K^+^ Ion Content Measurements

Proline content in leaf tissue was determined using a method described by Bates et al. (1973). The mixture containing 2 ml of sample supernatant, 2 ml of acetic acid and 2 ml of 2.5% acid Ninhydrin was boiled for 30 min, and the absorbance was determined at A520. The MDA content was determined according to Quan et al. (2004).

Na^+^ and K^+^ ion content was determined as described by Storey (1995). The dried leaves and roots were digested with 1 M HCl at 60°C for 1 h, and Na^+^, K^+^ contents were determined using atomic absorption spectrometry (PXSJ-216, Shanghai, China).

### Saline Field Trial of Transgenic Cotton

The three T_4_ transgenic cotton lines expressing *AvDH1* (08-66, 08-87, and 08-26) and the WT plants were field-tested at the Halophyte Garden Experimental Farm in Dongying (37°26′N, 118°40′E), the Yellow River Delta of China, in 2013 and 2014. The climate of the experimental area is temperate and monsoonal. The rainfall is variable with a greater distribution in July and August. The experiment was conducted in two nearby cotton fields. Analysis of soils (0–20 cm) sampled in early spring showed that both fields had sandy loam soils with similar contents of organic matter and main nutrients, but different salinity levels (**Table 1**). The soils were underlain with a saline water (EC_w_ = 25–30 dS/m) table at a 2.0–2.5 m depth.

Cotton plants were tested under moderate and high soil salinity levels, and laid out in a completely randomized block design with three replicates for each level. The plot was comprised of six rows, and each row was 10 m long. The inter-row spacing was 0.86 m wide and inter-plant spacing was 0.24 m. The soil was subjected to excess irrigation to leach soluble salts with fresh water (3,000 m^3^ ha^-1^) 30 days before planting each year. After the water had fully infiltrated the soil, the plots received 1,200 kg ha^-1^ of a commercial compound fertilizer containing (by weight) 30% N and 35% P_2_O_5_. Thereafter, the soil was plowed and harrowed when its mellowness was physically acceptable. Plots were sown on 2 May 2013 and 30 April 2014. Six to eight seeds were manually sown per hill, and furrows were mulched with a plastic film. The germination rate was counted 15 days after sowing. All the planting rows had a north–south orientation. Throughout the growing season, plots were not irrigated in 2013 and 2014 due to sufficient rainfall. Standard agronomical practices were performed for weed control and pesticide application unless indicated otherwise. The dynamics of salinity in the plant root zone (0–20 cm) was also determined in 2013 and 2014. Soil salinity levels of the moderate- and high-salinity fields decreased to 5.7 and 6.0 dS m^-1^, respectively, after irrigation (leaching) in 2013 (**Figure 5**). They gradually increased after sowing and reached their second peaks about 60 days after sowing (DAS) because of evapotranspiration. They then decreased at 90 DAS because of heavy rainfall in July and August. Similar soil salinity dynamics were also observed in 2014. Salinity values at a certain time differed greatly between the two fields, with the highly saline field being much higher than the moderately saline field (**Figure 5**). On 28 September, yield components were evaluated. 30 uniform plants in each plot were selected to evaluate lint percentage.

### Statistical Analyses

All data were presented as mean ± standard deviation (SD). Comparisons between transgenic and WT plants were performed using Student’s *t*-test. A *P*-value of <0.05 was considered statistically significant. All statistical analyses were performed using DPS 7.05.

## Author Contributions

FS, JC designed and performed the work. JC drafted the work. SW, HL, SF revised it critically for important intellectual content. YZ, WW, MX, RY, FD took part in part of field experiments.

## Conflict of Interest Statement

The authors declare that the research was conducted in the absence of any commercial or financial relationships that could be construed as a potential conflict of interest. The reviewer Ing-Feng Chang and handling Editor Keqiang Wu declared their shared affiliation, and the handling Editor states that, nevertheless, the process met the standards of a fair and objective review.
