# Corrigendum: Overexpression of an *Apocynum venetum* DEAD-Box Helicase Gene (*AvDH1*) in Cotton Confers Salinity Tolerance and Increases Yield in a Saline Field

**DOI:** 10.3389/fpls.2016.01041

**Published:** 2016-07-13

**Authors:** Jie Chen, Sibao Wan, Huaihua Liu, Shuli Fan, Yujuan Zhang, Wei Wang, Minxuan Xia, Rui Yuan, Fenni Deng, Fafu Shen

**Affiliations:** ^1^State Key Laboratory of Crop Biology, College of Agronomy, Shandong Agricultural UniversityTaian, China; ^2^College of Life Science, Shanghai UniversityShanghai, China; ^3^Cotton Research Institute – Chinese Academy of Agricultural SciencesAnyang, China; ^4^Cotton Research Center, Shandong Academy of Agricultural SciencesJinan, China

**Keywords:** DEAD-box helicase, *AvDH1*, cotton, salinity, yield, field trial

Reason for Corrigendum:

In the Original Research article, the “W” and “P” lanes in the published version of Figure 1C were spliced into the figure rather than run out as part of a contiguous gel/blot, we have regenerated new, unprocessed images via further experimentation. The revised version of Figure has been provided below in the last page and modifications have been made to the corresponding sections in the main text.

**Figure 1 The Published version:**
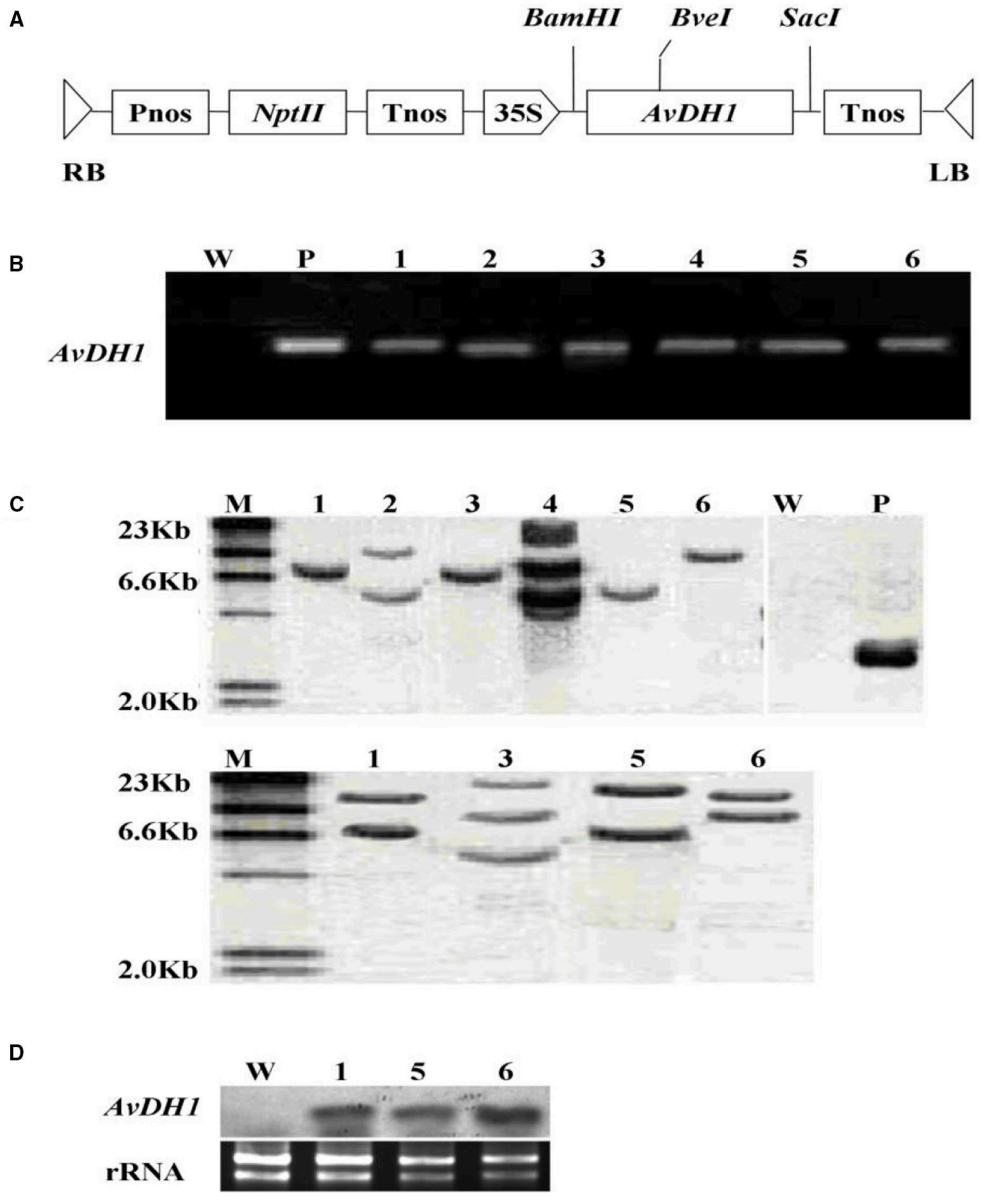
**Figure 1**.

**The revised version: Figure 1:**
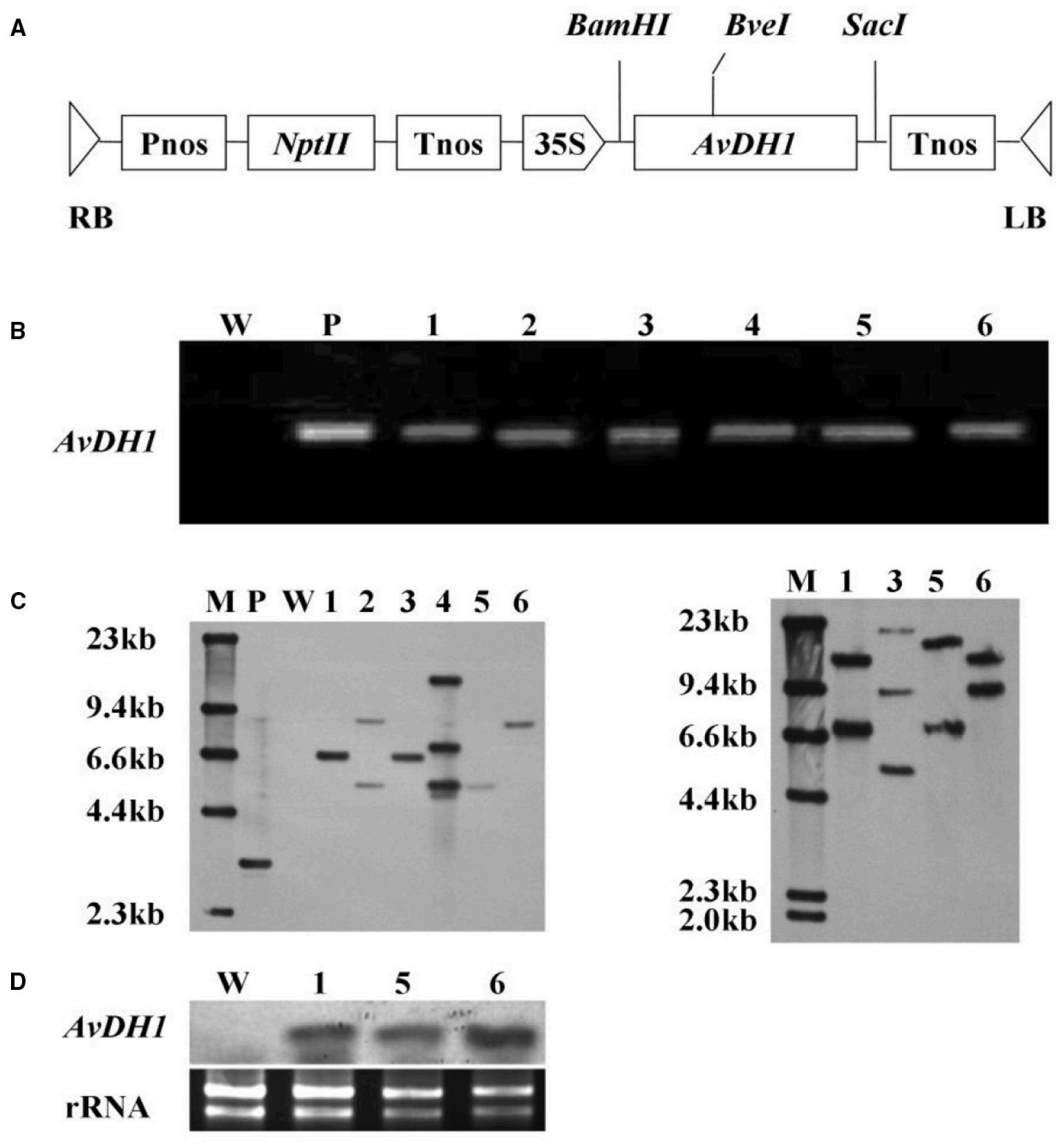
**Schematic structure of the T-DNA and molecular analysis of ***AvDH1***-expressing cotton. (A)** T-DNA region of the vector pBI121-*AvDH1*. RB, right T-DNA border; LB, left T-DNA border; Pnos, nopaline synthase gene promoter; *nptII*, neomycin phosphotransferase gene; Tnos, nopaline synthase gene terminator; 35 S, cauliflower mosaic virus 35 s promoter; *AvDH1, Apocynum venetum* DEAD-box helicase gene. **(B)** PCR analysis of genomic DNA from an untransformed control (W) and six independent T_0_ transgenic lines (1–6). P, pBI121-*AvDH1* as positive control. **(C)** Southern blot analysis of *AvDH1*-transformed cotton lines. Genomic DNA was digested with *Stu*I (left) and *Bve*I (right). The membrane was hybridized with a DIG-labeled *AvDH1* probe. M, molecular marker; P, pBI121-*AvDH1* as positive control; W, untransformed control; 1–6, transgenic lines 08–66, 08–89, 08–90, 08–92, 08–26, and 08–87, respectively. **(D)** Three independent T3 transgenic lines were confirmed by Northern blotting. Lower panel shows rRNA to confirm equal loading. 1, line 08–66; 5, line 08–26; 6, line 08–87.

In the “Materials & Methods” section, sub section “PCR and Southern Blot”, the last two sentences of the paragraph has been modified from

A [32P]-labeled AvDH1 gene was used as the probe. Southern blots were hybridized by following the standard procedure provided by the manufacturer. After hybridization and stringent washing, the radioactive membranes were exposed to an imaging plate (Fuji Photo Film, Japan) for 5 h or overnight to record the images.

to

A DIG-labeled marker (molecular weight marker II, Roche Diagnostics, Mannheim, Germany) was used for size estimation, and a digoxigenin (DIG)-labeled AvDH1 gene sequence was used as the probe. Incorporation of digoxigenin-11-dUTP into the AvDH1 probe was done by Taq DNA polymerase during a PCR reaction using forward primer AvDH1-F 5′-TTGGCGGCAATAGCGT-3′ and reverse primer AvDH1-R 5′-CCTTAGTAGCACCACCCT-3′, following the supplier's instruction (Roche Diagnostics). Southern blots were hybridized using a DIG-High Prime DNA Labeling and Detection Starter Kit II (Roche Diagnostics, Mannheim, Germany) following the standard procedure provided by the manufacturer. After hybridization and stringent washing, X-ray films (Biomax MS, Kodak) exposure was done for 0.5, 1, and 3 h to achieve the desired signal strength.

In the “Results” section, sub section “Regeneration and Analysis of Transgenic Cotton Plants”, the word “Upper” has been changed to “Left” and “lower” has been changed to “right”.

The authors apologize for the ambiguity. This error does not change the scientific conclusions of the article in any way.

## Conflict of interest statement

The authors declare that the research was conducted in the absence of any commercial or financial relationships that could be construed as a potential conflict of interest.

